# HTA and National Immunisation Program: an overview of competent bodies and of the impact of HTA on the decision-making process

**DOI:** 10.1093/eurpub/ckac129.327

**Published:** 2022-10-25

**Authors:** W Ricciardi

**Affiliations:** Department of Life Sciences and Public Health, Università Cattolica del Sacro Cuore, Rome, Italy

## Abstract

**Issue/problem:**

The WHO has recommended for many years to adopt transparent and standardized methods to decide upon the introduction of vaccines at national level. For this purpose, the Global Vaccine Action Plan has envisaged the establishment of National Immunization Technical Advisory Groups (NITAGs) to issue independent advice relied on the assessment of vaccines efficacy and safety and health technology assessment (HTA).

**Description of the problem:**

So far, 172 countries worldwide have a NITAG. Despite the growth in the number of NITAGs worldwide and their strengthening following process indicators identified by the WHO, the way how NITAGs issue recommendations and how they are considered in the development of vaccine policy still represent a matter to the attention of the scientific and policy community.

**Results:**

The evidence shows that assessment frameworks vary across countries. Furthermore, the legal basis and the authority, namely binding or advisory, recognised to them could influence the way how NITAGs work and impact on vaccine policy. Eventually, the final decision is also led by other aspects, including financial and contextual factors that are less standardizable but should be always taken into consideration in health policy. In fact, the final decision on the inclusion of a vaccination into the national immunization program is made by the Ministry of Health also based on the evaluation of HTA bodies working in the country.

**Lessons:**

Considering the central role of NITAGs in vaccine policy, some efforts should be done in order to make NITAGs more authoritative and collaborative. This could help, from one side, to make the relationship between final decision and NITAGs recommendations clearer and, on other one, to make it possible to have shared and timely evaluations. In this respect, Joint Clinical Assessment envisaged by the new EU regulation on HTA could prepare the way for a more transparent and timely vaccine policy at European level.

